# Low level ß-lactamase production in methicillin-resistant *staphylococcus aureus* strains with ß-lactam antibiotics-induced vancomycin resistance

**DOI:** 10.1186/1471-2180-12-69

**Published:** 2012-05-08

**Authors:** Yuriko Hirao, Yurika Ikeda-Dantsuji, Hidehito Matsui, Masaki Yoshida, Seiji Hori, Keisuke Sunakawa, Taiji Nakae, Hideaki Hanaki

**Affiliations:** 1 Laboratory for Antimicrobial Agents, Kitasato University, Tokyo, 108-8641, Japan; 2 Graduate School for Infection Control Sciences, Kitasato University, Tokyo, 108–8641, Japan; 3 Yamanashi Prefectural University, Kofu, 400-0062, Japan; 4 Department of Infectious Disease and Infection Control, Jikei University School of Medicine, Tokyo, 105-8461, Japan

**Keywords:** Antibiotic resistance, ß-lactam, Vancomycin, Staphylococcus aureus, ß-lactamase, MRSA

## Abstract

**Background:**

A class of methicillin-resistant *Staphylococcus aureus* (MRSA) shows resistance to vancomycin only in the presence of ß-lactam antibiotics (BIVR). This type of vancomycin resistance is mainly attributable to the rapid depletion of free vancomycin in the presence of ß-lactam antibiotics. This means that ß-lactam antibiotics remain active or intact in BIVR culture, although most MRSA cells are assumed to produce ß-lactamase. We hypothesised that the BIVR cells either did not harbour the ß-lactamase gene, *blaZ*, or the gene was quiescent. We tested this hypothesis by determining ß-lactamase activity and conducting PCR amplification of *blaZ*.

**Results:**

Five randomly selected laboratory stock BIVR strains showed an undetectable level of ß-lactamase activity and were *blaZ*-negative. Five non-BIVR stock strains showed an average ß-lactamase activity of 2.59 ± 0.35 U. To test freshly isolated MRSA, 353 clinical isolates were collected from 11 regionally distant hospitals. Among 25 BIVR strains, only 16% and 8% were *blaZ* positive and ß-lactamase-positive, respectively. In contrast, 95% and 61% of 328 non-BIVR strains had the *blaZ* gene and produced active ß-lactamase, respectively. To know the mechanism of low ß-lactamase activity in the BIVR cells, they were transformed with the plasmid carrying the *blaZ* gene*.* The transformants still showed a low level of ß-lactamase activity that was several orders of magnitude lower than that of *blaZ*-positive non-BIVR cells. Presence of the ß-lactamase gene in the transformants was tested by PCR amplification of *blaZ* using 11 pairs of primers covering the entire *blaZ* sequence. Yield of the PCR products was consistently low compared with that using *blaZ*-positive non-BIVR cells. Nucleotide sequencing of *blaZ* in one of the BIVR transformants revealed 10 amino acid substitutions. Thus, it is likely that the ß-lactamase gene was modified in the BIVR cells to downregulate active ß-lactamase production.

**Conclusions:**

We concluded that BIVR cells gain vancomycin resistance by the elimination or inactivation of ß-lactamase production, thereby preserving ß-lactam antibiotics in milieu, stimulating peptidoglycan metabolism, and depleting free vancomycin to a level below the minimum inhibitory concentration of vancomycin.

## Background

Individuals whose immune activity has been compromised by conditions, such as cancer, transplantation, blood dialysis, and aging often become infected with *Staphylococcus aureus*. Particularly problematic is infection by methicillin-resistant *S. aureus* (MRSA), for which antibiotic chemotherapy is often difficult and results in failure because this organism shows resistance to structurally and functionally diverse chemotherapeutic agents. Spread of MRSA was limited to hospital patients for a long period of time, but it has become more common in the broader community in recent years. Owing to the multi-antibiotic-resistant nature of MRSA, only a limited range of chemotherapeutic agents can be used; most commonly, vancomycin or the recently developed linezolid [1-3].

Vancomycin is a glycopeptide antibiotic with a molecular mass of 1449.3. It binds with the d-Ala-d-Ala terminals of the peptidoglycan structure and its precursors, and blocks the action of peptidoglycan transpeptidase or penicillin-binding proteins (PBPs), consequently inhibiting extension of the peptidoglycan network and growth of the cells [4,5]. Vancomycin is active against Gram-positive bacteria including enterococci and staphylococci [6]**,** whereas it is ineffective against Gram-negative bacteria, mainly because the outer membrane acts as a penetration barrier.

Another problem in MRSA-infected patients is co-infection with Gram-negative bacteria, such as *Pseudomonas aeruginosa*, which is naturally resistant to vancomycin and linezolid. One of the solutions for the chemotherapy of such mixed infections has been to use a combination of vancomycin and ß-lactam antibiotics [7]. In fact, this regime has been recommended for several decades in Japan and seems to be successful [8]. However, the use of this combination therapy has led to the emergence of MRSA that is resistant to vancomycin only in the presence of ß-lactam antibiotics, which is designated as BIVR [9,10].

BIVR is understood to have arisen because the use of ß-lactam antibiotics promotes peptidoglycan metabolism, probably due to partial ß-lactam-mediated damage of the peptidoglycan networks [11]. The affected cells upregulate the peptidoglycan biosynthetic pathways and repair systems, producing large amounts of peptidoglycan precursors, such as lipid-intermediate II with free d-Ala-d-Ala terminals [12,13]. Vancomycin tightly binds with the d-Ala-d-Ala structure of peptidoglycan and its intermediate precursors [4,5]. Consequently, the concentration of free vancomycin in milieu is lowered below its MIC and the cells begin to grow under such conditions [13].

The enzyme, ß-lactamase hydrolyses the ß-lactam ring of ß-lactam antibiotics and inactivates them, thereby rendering the cells resistant to ß-lactam antibiotics. *Staphylococcus* cells that have not been exposed to ß-lactam antibiotics do not possess the ß-lactamase gene, *blaZ*, and hence, are highly susceptible to ß-lactam antibiotics. However, clinical use of ß-lactam antibiotics enables the cells to harbour a plasmid bearing *blaZ* that encodes cell-associated penicillinase. These cells have two main emergency responses: one is to induce ß-lactamase and the other is to elicit the peptidoglycan recycling and repair system [14].

We generally assume that most MRSA cells are resistant to ß-lactam antibiotics owing mainly to the production of ß-lactamase [15] or of PBP2′ (or PBP2a) [16-18]. Therefore, ß-lactam antibiotics in MRSA culture are readily hydrolysed. However, the BIVR phenomenon is induced only in the presence of ß-lactam antibiotics, suggesting that ß-lactam antibiotics in culture remain intact. An empirical observation is that clinical isolates of BIVR cells seem to have a low level of ß-lactamase activity compared with that of non-BIVR MRSA. Accordingly, we hypothesised that ß-lactamase activity in BIVR cells was somehow downregulated, which prompted us to investigate the relationship between the BIVR phenomenon and ß-lactamase activity.

## Results

### Properties of the representative laboratory BIVR and non-BIVR cells

**Figure 1:**
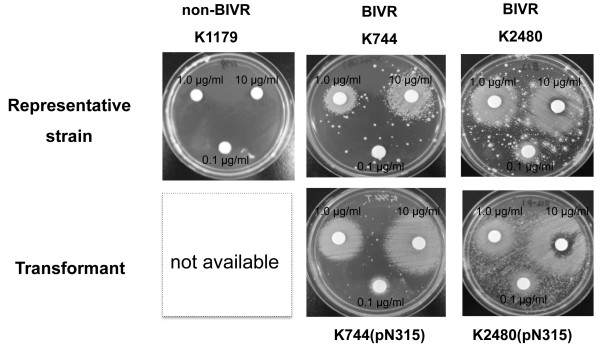
**BIVR test of the representative strains.** The class of MRSA and the strain number are shown in the figure. Upper panels show photographs of the representative strains and the lower panels show the respective transformants with the plasmid pN315. K1179 was a non-BIVR MRSA harbouring the *blaZ* gene and producing a high level of ß-lactamase. Hence, a transformant was not available. xx μg/ml denotes the concentration of ceftizoxime in the disk.

BIVR is a class of MRSA that is susceptible to vancomycin at ≤2 μg/ml, and becomes vancomycin-resistant in the presence of ß-lactam antibiotics. We tested our stock strains used in this study for the BIVR phenomenon. Strains Mu3, K101, K638, K670, K744 and K2480 were streaked on Mu3 agar plates impregnated with 4 μg/ml vancomycin. None of these strains grew on the plates, confirming that the BIVR cells were vancomycin-susceptible. The MICs of vancomycin for these strains were 1–2 μg/ml (Table 1). When ß-lactam impregnated disks with concentrations of 0.1, 1.0 and 10 μg/ml ceftizoxime (Astellas Pharma, Tokyo, Japan) were placed on the plates, all the strains grew around the disks (Figure 1, only K744 and K2480 are shown). Thus, the BIVR property in the laboratory stock strains was confirmed.

**Table 1:** MIC of antibiotics in the strains used in this study (μg/ml)

**Strain**	**MPIPC**	**IPM**	**VAN**	**LZD**	**ABPC**	**ZOX**	**CAZ**
Reference strains							
FDA209P (MSSA)	0.5	≤0.25	0.5	2	0.25	4	16
N315 (non-BIVR)	32	1	0.5	2	32	>128	128
Mu3 (BIVR)	>128	64	2	2	32	>128	>128
Lab. stock non-BIVR							
K1	>128	128	2	2	64	>128	>128
K27	>128	64	2	2	64	>128	>128
K51	>128	128	2	2	64	>128	>128
K54	>128	64	1	2	64	>128	>128
K1179	64	16	1	2	32	>128	>128
Lab. stock BIVR							
K101	>128	64	2	2	32	>128	>128
K638	>128	128	2	2	32	>128	>128
K670	>128	128	2	2	32	>128	>128
K744	>128	128	1	2	16	>128	>128
K2480	>128	64	1	2	32	>128	>128
Transformants							
K744-T	>128	>128	1	1	16	>128	>128
K2480-T	>128	128	0.5	2	32	>128	>128

*MPIPC*, oxacillin; *IPM*, imipenem; *VAN*, vancomycin; *LZD*, linezolid; *ABPC*, ampicillin; *ZOX*, ceftizoxime; *CAZ*, ceftazidime.

Cells classified as non-BIVR MRSA, which were the K1, K27, K51,K54, K1179 and N315 strains, were tested similarly. These cells were vancomycin-susceptible and did not grow on the vancomycin-containing plates in the presence or absence of ß-lactam-impregnated disks (Figure 1, K1179). The MICs of vancomycin for these strains were 0.5–2 μg/ml.

### ß-lactamase activity in BIVR and non-BIVR cells

Based on our hypothesis that BIVR cells might express a low level of ß-lactamase, we compared the enzyme activity in five laboratory stock non-BIVR and BIVR strains. The ß-lactamase activity in non-BIVR strains ranged from 0.127 to 11.1 U (Table 2) with an average value of 2.59 ± 0.35 U, while that in all five BIVR strains showed an undetectable level of ß-lactamase, <10^–4^ U. Thus, it became evident that ß-lactamase activity in BIVR cells was at least three orders of magnitude lower than that in non-BIVR cells. The following explanations are offered: (i) the non-BIVR cells harboured a plasmid bearing the ß-lactamase gene (*blaZ*), but the BIVR cells did not; or (ii) both BIVR and non-BIVR cells harboured a *blaZ*-bearing plasmid, but the production of active ß-lactamase in BIVR cells was suppressed or downregulated.

**Table 2:** **β-Lactamase activity and presence of*blaZ*in laboratory-stock BIVR and non-BIVR strains**

**Strains**	***blaZ***	**β-lactamase activity (μmol/min/mg protein)**
Reference strains		
FDA209P (MSSA)	-	<1 × 10^-4^
N315 (non-BIVR)	+	7.39 × 10^-1^
Mu3 (BIVR)	-	<1 × 10^-4^
BIVR strains		
K101	-	<1 × 10^-4^
K638	-	<1 × 10^-4^
K670	-	<1 × 10^-4^
K744	-	<1 × 10^-4^
K2480	-	<1 × 10^-4^
Non-BIVR strains		
K1	+	1.94 × 10^-1^
K27	+	1.27 × 10^-1^
K51	+	6.24 × 10^-1^
K54	+	11.1
K1179	+	9.06 × 10^-1^
Transformants		
K744-T	+	<1 × 10^-4^
K2480-T	+	<1 × 10^-4^

**Figure 2:**
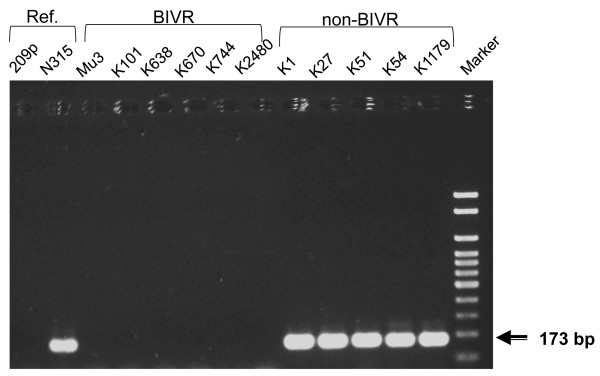
**Agarose gel electrophoretograms of the PCR product.** Primer K was used for the PCR of *blaZ* and the conditions for the thermal cycler setting are given in the text. A fixed agarose concentration (2%) was used. The gel was stained with GelRed and visualised under UV light. Marker, LowRange 100 bp DNA markers; FDA209P, negative control; N315, positive control; the MRSA class and strain number are shown in the figure.

To test for the presence of the ß-lactamase gene, *blaZ* was amplified by PCR using a primer set K shown in Table 3. N315 and FDA209P cells were used as positive and negative references, respectively*.* As seen in Figure 2, the PCR products amplified from N315 cells showed a large distinct band with nucleotide numbers corresponding to about 170 bp, which was the expected PCR product. The PCR product was undetectable when the FDA209P DNA was used as a template. Similarly, PCR was carried out using the template DNA from Mu3, K101, K638, K670, K744 and K2480 cells and no detectable band was found (Figure 2). The results suggested that these BIVR strains did not have the ß-lactamase gene, which was fully consistent with the finding of undetectable ß-lactamase activity. In contrast, PCR experiments using the DNA template from non-BIVR strains showed clear bands corresponding to the expected *blaZ* product. These results were again consistent with that of the ß-lactamase assay and with the above explanation (i); whether or not BIVR cells possessed the gene encoding ß-lactamase, but did not give the answer to the above question (ii); whether the expression of the ß-lactamase gene in BIVR could be suppressed. Therefore, the following experiments were conducted.

**Table 3:** Primer sets used

**Code**	**Nucleotide sequence**
A	(F) 5’-GGTTGCTGATAAAAGTGGTCAA-3’ (R) 5’-CTCGAAAATAATAAAGGGAAAATCA-3’
B	(F) 5’-AAGAAATCGGTGGAATCAAAAA-3’ (R) 5’-GTTCAGATTGGCCCTTAGGA-3’
C	(F) 5’-TTGCCTATGCTTCGACTTCA-3’ (R) 5’-GCAGCAGGCGTTGAAGTATC-3’
D	(F) 5’-TCAAACAGTTCACATGCCAAA-3’ (R) 5’-TTTTTGATTCCACCGATTTCTT-3’
E	(F) 5’-GCCATTTTGACACCTTCTTTC-3’ (R) 5’-CGAAGCATAGGCAAATCTCTT-3’
F	(F) 5’-TGAGGCTTCAATGACATATAGTGATAA-3’ (R) 5’-GTTCAGATTGGCCCTTAGGA-3’
G	(F) 5’-TGTTTAATAATAAAAACGGAGACACTT-3’ (R) 5’-TCAACTTATCATTTGGCTTATCACTT-3’
H	(F) 5’-AAGAAATCGGTGGAATCAAAAA-3’ (R) 5’-TTTAAAGTCTTGCCGAAAGCA-3’
I	(F) 5’-AAGAAATCGGTGGAATCAAAAA-3’ (R) 5’-TCGAAAATAATAAAGGGAAAATCA-3’
J	(F) 5’-GCCATTTTGACACCTTCTTTC-3’ (R) 5’-AGCAGCAGGCGTTGAAGTAT -3’
K*	(F) 5’-ACTTCAACACCTGCTGCTTTC-3’ (R) 5’-TGACCACTTTTATCAGCAACC-3’

* Primer K was from reference [19].

F and R denote the forward and reverse sequences, respectively.

**Figure 3:**
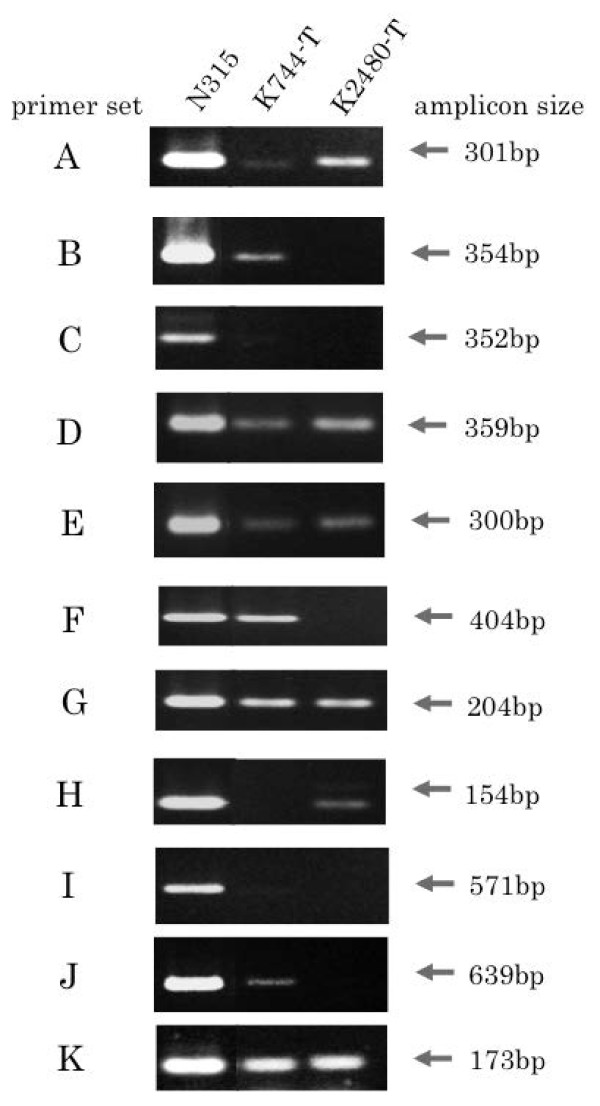
**PCR products of the*blaZ*gene.** The primer sets in alphabetical order correspond with that in Table 2. Agarose gel electrophoresis was carried out as described in the legend to Figure 2. Only a part of the electrophoretogram is shown. Arrow and bp, the amplicon size; N315, K744-T and K2480-T were the source of the template DNA.

Codes correspond with that in Figure 3.

### Transformation of BIVR cells with a plasmid bearing the ß-lactamase gene

To test whether the ß-lactamase gene was stable in the BIVR cells and expressed active enzyme, the BIVR cells were transformed with the plasmid carrying *blaZ.* Plasmid DNA was extracted from N315 cells (bearing the pN315 plasmid) cultured in 5.0 ml brain–heart infusion broth and purified by the Plasmid Mini kit (Qiagen, Tokyo, Japan). The average yield of DNA appeared to be ~50 ng. To confirm that the extracts contained the plasmid bearing the ß-lactamase gene, they were subjected to PCR amplification using the primer set K. Agarose gel electrophoresis clearly showed a single distinct large band corresponding to the size of the expected PCR product (similar to the result in Figure 2, Ref. N315). Attempts have been made to extract the plasmid DNA from BIVR cells, such as K744 and five other strains, but the yield was consistently undetectable except for the K2480 cells, which showed a trace amount of DNA. PCR amplification of *blaZ* taking the K2840 extracts as the template yielded no visible band*.*

The BIVR cells, K744 and K2480, were transformed with plasmid DNA extracted from N315 cells. Selection of the transformants for ß-lactam resistance was difficult because the recipient cells were ß-lactam-resistant beforehand to a certain extent. Thus, transformants were selected on agar plates impregnated with a 1.5-fold MIC equivalent of ampicillin and obtained from K744 and K2480 strains (K744-T and K2480-T, respectively). Presence of the *blaZ* gene in the K744-T and K2480-T cells was confirmed by PCR using whole-cell extracts as the template, and subsequent agarose gel electrophoresis yielded a single DNA band corresponding to that obtained from N315 cells (Figure 3). Note that the amount of PCR products using K744-T and K2480-T DNA as the template appeared low compared with that from N315 cells (Figure 3). The identity of untransformed and transformed cells was confirmed by pulse-field gel electrophoresis of the chromosomal DNA treated with *Sma*I. Unsuccessful attempts were made to transform FDA209P with the pN315 plasmid. The reasons for failure of this transformation experiment remain obscure.

ß-lactamase activity was determined using N315, K744-T and K2480-T cells. The results showed that activity in N315 cells appeared to be 0.74 U, while the levels in K744-T and K2480-T cells were undetectable (Table 2). Plasmid DNA from K744-T was undetectable, but a trace amount was extracted from K2480-T comparable with the level from the untransformed parent cells. Attempts have been made to amplify the *blaZ* DNA using the column eluate of the extracts as the template. The PCR product using the K744-T extracts was undetectable and that of K2480-T showed a faint band, which was identical with the control experiment using untransformed K2480. We interpreted these results to mean that the BIVR cells might have a mechanism to modify the ß-lactamase gene. The transformants were subjected to the BIVR test. K744-T and K2480-T cells showed a strong BIVR reaction in the presence of 0.1, 1.0 and 10 μg/ml ceftizoxime (Figure 1), confirming that the BIVR property was unchanged even in the presence of modified *blaZ*.

### Search for mutations in the *blaZ* gene of the transformants

**Figure 4:**
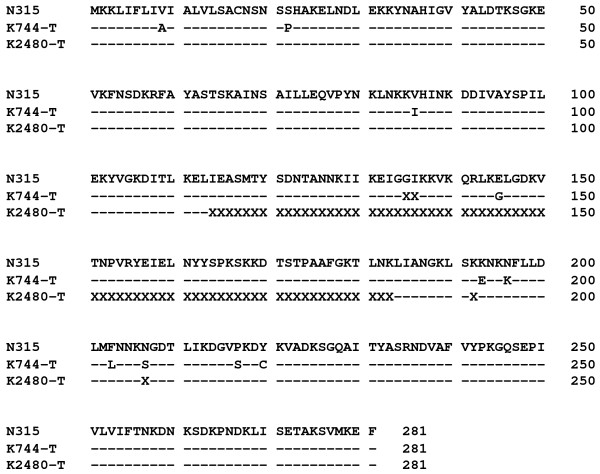
**Amino acid sequence of the*blaZ*gene in the transformant.** The *blaZ* gene in the transformants K744-T and K2480-T as well as that of the donor plasmid pN315 was amplified by PCR using the primer pairs listed in Table 2. The nucleotide sequence was determined by the dideoxy chain termination method at Nippon Gene Research Laboratories (Miyagi, Japan). The nucleotide sequence was aligned by the computer programme, DNASIS Pro (Hitachi Software Engineering Co., Ltd., Tokyo, Japan), and was converted to the amino acid sequence. Amino acids are expressed by a single letter code. X mark denotes the amino acid residue, which could not be specified in this study. – denotes the amino acid residue, which is identical to that of pN315.

One of the possibilities for low ß-lactamase activity in the BIVR transformants could be that the ß-lactamase gene could have mutations or is somehow modified. Experiments were carried out to amplify and sequence *blaZ* using 11 different pairs of primers (Table 3) covering the entire *blaZ* sequence. As K744-T DNA or K2480-T DNA was used as a template, the yield of PCR product was consistently low in all the experiments (Figure 3). However, attempts were made to determine their nucleotide sequences comparing with the sequence from pN315 (the *blaZ* sequence in our experiments appeared identical to that of the database)*.* Nucleotide sequencing of the PCR products from the K744-T template showed 10 amino acid substitutions at Val9Ala, Ser22Pro, Val86Ile, Glu145Gly, Lys193Glu, Asn196Lys, Phe203Leu, Asn207Ser, Pro217Ser and Tyr220Cys compared with the *blaZ* sequence on pN315 (Figure 4)*.* Nucleotide sequencing of the products using the K2480-T templates could not be completed owing to the poor yield of PCR products (Figure 3). Therefore, it is not clear whether or not *blaZ* in K2840-T had mutations. However, it was strongly suggested that *blaZ* in K2480-T was modified because the amount of PCR product was consistently low or undetectable in some cases using 11 different pairs of primers, compared with the amount of PCR product from N315 cells (Figure 3).

Taken together, these findings indicated that introduction of the *blaZ* gene into BIVR cells did not elevate the ß-lactamase activity nor had much influence on the BIVR property, which might have been due to modification of the *blaZ* gene in the transformants. Therefore, these findings support the prediction that the ß-lactamase gene was downregulated or modified in BIVR cells.

### ß-lactamase activity and occurrence of the *blaZ* gene in clinical isolates of BIVR and non-BIVR MRSA

The above results were obtained using laboratory stock strains. To determine whether a similar tendency would be seen in fresh clinical isolates, we collected a total of 353 strains of independently isolated MRSA from 11 regionally distant hospitals. Twenty-five strains were classified as BIVR, which was equivalent to 7.0% of the total, while 328 strains (92.9%) were non-BIVR. All these strains were subjected to the *blaZ* test by PCR and a qualitative ß-lactamase test using a nitrocefin-impregnated disk. Among the 25 BIVR strains, 21 (84.0%) were *blaZ*-negative and 23 (92.0%) yielded negative results for the nitrocefin test (Table 4). Among the non-BIVR strains, 310 (94.5%) were *blaZ*-positive and only 18 (5.5%) were *blaZ*-negative. Similarly, 223 strains (61.0%) yielded positive results for the nitrocefin test and the remaining 128 (39.0%) gave negative results (Table 4). A statistically significant difference in the occurrence of the *blaZ* gene and ß-lactamase activity between the BIVR and non-BIVR strains was found with a probability <0.01 by the χ^2^ and Fisher’s tests. These results clearly showed a trend for BIVR cells to lack the ß-lactamase gene and not produce active ß-lactamase, whereas most non-BIVR cells possessed the *blaZ* gene and a significant fraction (61.0%) produced ß-lactamase. It should be noted that the nitrocefin test is a qualitative assay and might not be sensitive enough to detect low levels of ß-lactamase. To investigate this possibility, we randomly selected 10 non-BIVR strains that were *blaZ*-positive and -negative for the nitrocefin test and carried out a quantitative ß-lactamase assay. All cells produced a low level of ß-lactamase ranging from 2.74×10^–3^ to 2.1×10^–2^ U with an average of 7.25×10^–3^ ± 1.25×10^–2^ U (Table 5). Therefore, the number of ß-lactamase-positive strains must be much higher.

**Table 4:** **Presence of*blaZ*gene and β-lactamase activity in clinical isolates of BIVR and non-BIVR strains**

	***blaZ***		**Nitrocefin test**	
	**+**	**-**	**+**	**-**
BIVR	4 (16.0%)	21 (84.0%)	2 (8.0%)	23 (92.0%)
Non-BIVR	310 (94.5%)	18 (5.5%)	200 (61.0%)	128 (39.0%)

**Table 5:** **Quantitative β-lactamase activity, nitrocefin test and presence of*blaZ*in randomly selected clinical isolates of BIVR and non-BIVR**

**Phenotype**	***blaZ***	**Nitrocefin test**	**ß-lactamase (μmol/min/mg protein)**	
			**Range**	**Average ± STD**
BIVR (n = 5)	**-**	**-**	<1 × 10^-4^	<1 × 10^-4^
Non-BIVR (n = 10)	**+**	**+**	1.03 × 10^-3^ – 4.48	0.79 ± 1.84
Non-BIVR (n = 10)	**+**	**-**	2.76 × 10^-4^– 2.13 × 10^-2^	7.28 × 10^-3^ ± 1.25 × 10^-2^

Ten randomly selected non-BIVR strains that were *blaZ-*positive and positive for the nitrocefin test were subjected to the quantitative ß-lactamase assay. The activity ranged from 0.103 to 0.103×10^–3^ U with an average of 0.79 ± 1.84 U. Thus, it is likely that most non-BIVR cells produced ß-lactamase. Activity in BIVR cells (*blaZ*-negative and nitrocefin-test-negative) was undetectable.

## Discussion

This paper addressed the question of whether BIVR cells possess the *blaZ* gene and produce active ß-lactamase because BIVR cells show resistance to vancomycin only in the presence of ß-lactam antibiotics. This means that ß-lactam antibiotics must remain active in the BIVR milieu. Tests using laboratory stock strains revealed that all BIVR cells lacked *blaZ* and showed an undetectable level of ß-lactamase activity. All the laboratory stock non-BIVR cells possessed *blaZ* and produced high levels of ß-lactamase. This trend was confirmed using 353 clinical isolates including 25 BIVR and 325 non-BIVR strains. Transformation of the BIVR cells with a plasmid bearing *blaZ* revealed that: (i) ß-lactamase activity was undetectable; (ii) an attempt to extract the plasmid bearing *blaZ* was unsuccessful; (iii) PCR amplification of *blaZ* yielded a very low level of products in all 11 experiments using 11 different primer sets; and (iv) the nucleotide sequence of the PCR products using the K744-T template revealed 10 amino acid substitutions.

A plausible explanation of the results is that a low or undetectable level of ß-lactamase in BIVR cells enables ß-lactam antibiotics to remain active, thereby promoting peptidoglycan metabolism and the repair system producing large amounts of peptidoglycan precursors with unbound d-Ala-d-Ala terminals [4,5]. The precursors bind with free vancomycin, lowering the vancomycin concentration in milieu below the MIC of vancomycin. The BIVR cells begin to grow under these conditions, resulting in vancomycin resistance.

In the presence of ß-lactam antibiotics, a bacterial cell probably detects the peptidoglycan fragments generated by the ß-lactam action and might respond by producing ß-lactamase or promoting the peptidoglycan biosynthetic cascade and repair system [14]. Switching from one response to the other is assumed to be regulated by the balance of two peptidoglycan intermediates, such as anhMurNAc-tripeptide and UDP-MurNac-pentapeptide; a scenario reported in *Escherichia coli*[14].

If this scenario is applicable to *S. aureus* cells, BIVR and non-BIVR may be explained as follows. In the presence of ß-lactam antibiotics, MRSA cells, which have cryptic mutations to promote peptidoglycan metabolism, produce large amounts of peptidoglycan intermediates and deplete free vancomycin. *S. aureus* responding in this way may be BIVR. In contrast, in the presence of ß-lactam antibiotics, MRSA cells with a wild-type level of peptidoglycan metabolism undergo activation of the ß-lactamase-producing pathway. They may be the vancomycin-susceptible non-BIVR MRSA. However, this interpretation does not explain the discovery reported in this study that BIVR cells tend to exclude the plasmid bearing the ß-lactamase gene, and downregulate the production of active ß-lactamase, probably modifying the *blaZ* gene. These observations may be accounted for by suggesting that BIVR cells exclude *blaZ* or do not produce active ß-lactamase to maintain intact ß-lactam antibiotics in milieu to promote peptidoglycan metabolism. If ß-lactam antibiotics are hydrolyzed by the ß-lactamase, upregulated peptidoglycan metabolism would cease and the cell would be killed immediately by vancomycin.

**Figure 5:**
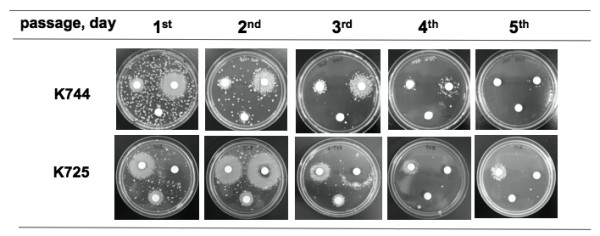
**Phase-out of the BIVR phenomenon.** BIVR cells were transferred to antibiotic-free MH agar and the plate was incubated at 37°C for 24 h. Cell suspensions from the plates were inoculated again on antibiotic-free MH agar and incubated for 24 h at 37°C. This serial transfer and culture was continued for 5 consecutive days. The culture was subjected to the BIVR test every day. Only a representative strain from the laboratory stock BIVR, K744 and freshly isolated clinical strains, K725, is presented. 1st to 5th represents the cycle of passage in the antibiotic-free medium.

To find a solution for the treatment of BIVR infection, we conducted serial passage experiments of BIVR cells in an antibiotic-free medium for several consecutive days and tested the fate of the BIVR cells. Figure 5 shows the BIVR test of the cells subjected for serial passage in the antibiotic-free medium. For the sake of space, only one strain each of the laboratory stock BIVR (K744) and freshly isolated clinical BIVR (K724) was presented. The BIVR cell properties were phased out by 5 consecutive days of passages. These cells, whose BIVR properties were gradually phased out, showed the non-BIVR phenotype when subjected to the BIVR test again. The mechanism of phasing out was not investigated further. The lesson from this experiment is that, once BIVR cells are isolated from patients, the use of ß-lactam antibiotics should be terminated for a while until the BIVR cells are phased out, and another type of antibiotic effective against Gram-negative bacteria should be used.

## Conclusions

A class of *S. aureus*, which shows vancomycin resistance only in the presence of ß-lactam antibiotics (BIVR), was tested for the presence of the ß-lactamase gene (*blaZ*) by PCR, and for the production of active ß-lactamase. The rationale for this study was that ß-lactam antibiotics in BIVR culture must be preserved to induce vancomycin resistance. However, it is generally assumed that the majority of MRSA strains harbour a plasmid bearing *blaZ* and produce active ß-lactamase. Five randomly selected laboratory stock BIVR strains showed no trace of either *blaZ* or ß-lactamase activity, whereas five non-BIVR laboratory strains possessed *blaZ*, and produced ß-lactamase at an average level of 2.59 U. Among 353 strains of freshly isolated MRSA, 25 and 325 were BIVR and non-BIVR, respectively. Of the 25 BIVR strains, only four (16%) and two (8%) strains were *blaZ*-positive and yielded a positive result for the ß-lactamase test, respectively. Among the non-BIVR strains, 310 (94.5%) and >200 (>61%) were *blaZ-*positive and yielded a positive result for the ß-lactamase test. Transformation of BIVR cells with a plasmid bearing *blaZ* still showed an undetectable level of ß-lactamase activity that probably was due to modification of the transformed *blaZ* gene. These results clearly demonstrate that the majority of BIVR cells do not produce active ß-lactamase either by avoiding harbouring of the *blaZ* gene or by modification of *blaZ* that preserves ß-lactam antibiotics in the BIVR milieu and induces vancomycin resistance.

## Methods

### Bacterial strains used and culture conditions

The bacterial strains used for antibiotic-susceptible *S. aureus* and a representative BIVR strain were FDA209P and Mu3 [20], respectively. The MICs of vancomycin in FDA209P and Mu3 were 0.5 μg/ml and 2 μg/ml, respectively, and those of ceftizoxime were 4 μg/ml and >128 μg/ml, respectively (Table 1). Representative BIVR and non-BIVR strains from this laboratory were K744 and K1179, respectively, and their properties have been reported previously [13]. Four additional strains each of BIVR and non-BIVR were also used. N315 is a strain harbouring plasmids that bear the ß-lactamase gene as reported previously [21], and was used as the source of the ß-lactamase gene. A total of 353 strains of MRSA were collected from clinical sources and subjected to the BIVR and ß-lactamase tests. Culture media used were Mueller–Hinton (MH) broth (Becton–Dickinson, Tokyo, Japan), Mu3 agar (Becton–Dickinson) and MH agar (Becton–Dickinson), depending on the purpose, and cells were incubated at 35°C for the desired period of time.

### BIVR test

BIVR was defined according to an earlier report [10,22]. Briefly, MRSA cells were grown in MH broth overnight at 35°C in the presence of 1 μg/ml ceftizoxime and 0.1 ml aliquots of cell suspensions adjusted to A_578_^1cm^ = 0.3 was streaked on an Mu3 agar plate impregnated with 4 μg/ml vancomycin. An 8-mm paper disk impregnated with 80 μl 0.1, 1.0 or 10.0 μg/ml ceftizoxime was placed on the agar plate. Cells showing a growth zone around the disk were judged to be BIVR.

### PCR

The *blaZ* genes encoding ß-lactamase were amplified by PCR with the following thermal cycler settings: 98°C for 30 s for the initial denaturation and then 30 cycles of denaturation, annealing and extension at 98°C for 5 s, 57°C for 10 s and 72°C for 10 s, respectively. A primer pair used for *blaZ* detection is listed in Table 2. Phusion DNA polymerase (Finzymes, Espoo, Finland) was used. The PCR products were analysed by agarose gel electrophoresis and visualised by staining with GelRed (Biotim Hayward, CA, USA). The marker used was LowRange 100bp DNA ladder marker (Norgen Bioteck Corp, Toronto, Canada).

### Determination of ß-lactamase activity

Beta-lactamase activity was determined either by the paper disk or spectrophotometric method [23]. For the semi-quantitative assay, an 8-mm paper disk impregnated with 80 μl 550 μg/ml nitrocefin was placed on colonies on the agar plate. The cells that developed a pink to red colour within 30 min were judged to be ß-lactamase-positive. The quantitative ß-lactamase assay was carried out as follows. Bacterial cells were grown overnight at 37°C in a 4-ml L-broth supplemented with 10 μg/ml ceftazidime with shaking at 250 rpm, and were harvested by centrifugation at 10 000 × *g* for 10 min. Pellets were washed once with a 4-ml aliquot of 50 mM sodium phosphate buffer, pH 7.2, containing 145 mM sodium chloride, and were suspended in 400 μl of the same buffer. Over 99% of the ß-lactamase was associated with the centrifuged cell pellets, and therefore the assay was carried out using the washed cell suspension. A pair of 1.0-ml reaction mixtures was prepared containing 10 μl cell suspension, 10 μl 100 mM EDTA and 880 μl 50 mM sodium phosphate buffer, pH 7.0. The reaction was initiated by adding 100 μl 500 μM nitrocefin, and one tube was incubated for 3 min and the other for 13 min. The tubes were centrifuged at 12 000 × *g* for 2 min, and clear supernatant was separated. A_486_ was determined at 5 and 15 min. Reaction velocity per minute was calculated by subtracting A_486_ at 5 min from that at 15 min divided by 10. Colour development from 5 to 15 min appeared linear under the conditions. For the cells with low ß-lactamase activity, 100 μl cell suspension was used and incubated at 24°C for 30 min. One unit of the enzymatic activity was defined as μmol nitrocefin hydrolysis/min/mg protein.

### Quantification of cellular protein

Cell suspensions were mixed with 2.0% of sodium dodecyl sulphate, and the mixture was heated at 100°C for 5 min and then centrifuged at 12 000 × *g* for 5 min. Protein concentration in the clear supernatant was determined using the BioRad Protein Assay kit (BioRad, Hercules, CA, USA) according to the manufacturer’s instructions.

### Determination of MIC of antibiotics

The MIC of antibiotics was determined by the agar dilution method according to the Clinical and Laboratory Standards Institute manual [24].

### Extraction of plasmid DNA

Bacterial cells were grown overnight in 5.0 ml brain–heart infusion broth (Becton–Dickinson) containing 10 μg/ml ceftizoxime, and harvested by centrifugation at 6000 × *g* for 10 min. Cells were treated with 50 μg/ml lysostaphin at 37°C for 40 min. Plasmid DNA was extracted using the Qiagen Plasmid Mini kit, according to the manufacturer’s instructions. DNA was analysed by agarose gel electrophoresis (1.0%), stained with GelRed and visualised under UV light.

### Transformation experiments

Transformation-competent cells were prepared according to the manufacturer’s instructions of the MicroPulser (BioRad). Transformation experiments were carried out using 250 ng DNA and the MicroPulser according to the manufacturer’s instructions. Transformants were selected on agar plates impregnated with a 1.5-fold MIC equivalent of ampicillin.

### Statistical analysis

The χ^2^ and Fisher’s tests were carried out using a computer programme embedded in Microsoft Excel.

## Abbreviations

MRSA, Methicillin-resistant Staphylococcus aureus; PBP, Penicillin binding protein; BIVR, ß-lactam antibiotic-induced vancomycin-resistant MRSA; blaZ, Gene encoding ß-lactamase; MIC, Minimum inhibitory concentration.

## Authors’ contributions

YH carried out the PCR experiments, ß-lactamase assay and the BIVR test. YI-D contributed to the nucleotide sequencing and the pulse-field gel electrophoresis. HM carried out computer-aided nucleotide and amino acid alignments. MY, SH and KS contributed to the collection of clinical isolates of MRSA. TN consulted with the investigators on the data acquisition and wrote the draft paper. HH conducted this study and gave final approval of the version of the paper to be submitted. All authors read and approved the final manuscript.
